# Involvement of Bcl-xL in Neuronal Function and Development

**DOI:** 10.3390/ijms22063202

**Published:** 2021-03-21

**Authors:** Julie Bas, Trang Nguyen, Germain Gillet

**Affiliations:** 1 Centre Léon Bérard, Centre de Recherche en Cancérologie de Lyon, Université de Lyon, Université Claude Bernard Lyon 1, INSERM 1052, CNRS 5286, 69008 Lyon, France; julie.bas@outlook.fr (J.B.); trangnguyen239@gmail.com (T.N.); 2 Hospices Civils de Lyon, Laboratoire d’anatomie et Cytologie Pathologiques, Centre Hospitalier Lyon Sud, Chemin du Grand Revoyet, 69495 Pierre Bénite, France

**Keywords:** Bcl-xL, apoptosis, neurons, mitochondria, endoplasmic reticulum, caspases

## Abstract

The B-cell lymphoma (Bcl-2) family of proteins are mainly known for their role in the regulation of apoptosis by preventing pore formation at the mitochondrial outer membrane and subsequent caspase activation. However, Bcl-2 proteins also have non-canonical functions, independent of apoptosis. Indeed, the cell death machinery, including Bcl-2 homologs, was reported to be essential for the central nervous system (CNS), notably with respect to synaptic transmission and axon pruning. Here we focused on Bcl-xL, a close Bcl-2 homolog, which plays a major role in neuronal development, as *bclx* knock out mice prematurely die at embryonic day 13.5, showing massive apoptosis in the CNS. In addition, we present evidence that Bcl-xL fosters ATP generation by the mitochondria to fuel high energy needs by neurons, and its contribution to synaptic transmission. We discuss how Bcl-xL might control local and transient activation of caspases in neurons without causing cell death. Consistently, Bcl-xL may contribute to morphological changes, such as sprouting and retractation of axon branches, in the context of CNS plasticity. Regarding degenerative diseases and aging, a better understanding of the numerous roles of the cell death machinery in neurons may have future clinical implications.

## 1. Introduction

**Figure 1:**
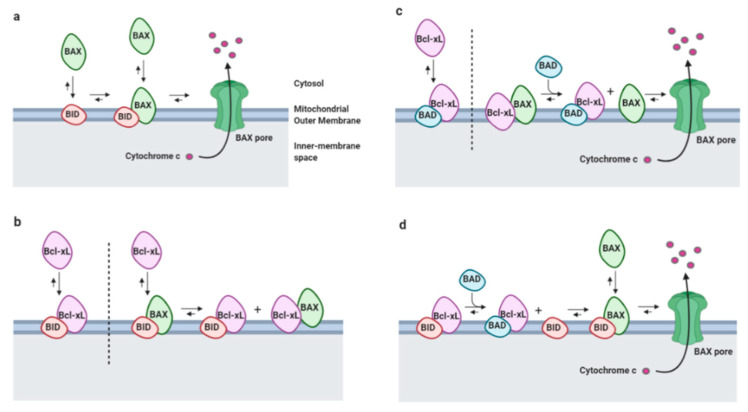
The interactions between the different members of the B-cell lymphoma (Bcl-2) family control mitochondria outer membrane permeabilization (MOMP) and subsequent apoptosis. All interactions are reversible, the overall equilibrium depends on affinity changes, which are affected by interactions with the lipid bilayer. (**a**) Bid is a BH3-only protein known as an “activator”, which triggers the formation of Bak/Bax oligomers when it interacts with Bax at the mitochondria outer membrane (MOM), subsequently leading to the formation of “pores” resulting in the release of Cytochrome c. (**b**) Once inserted into the membrane, Bid and Bax can recruit anti-apoptotic proteins such as Bcl-xL, resulting in mutual sequestration and inhibition of both pro- and anti-apoptotic proteins. Bcl-xL prevents both Bid from activating Bax and the oligomerization of Bax, thus inhibiting MOMP. (**c**,**d**) The BH3-only Bad protein is called “sensitizer” because it indirectly induces MOMP by binding to Bcl-xL. Since Bcl-xL has a higher affinity for Bid than Bax, an increase in Bad levels will first release Bax upstream of Bid activation, resulting in subsequent MOMP. Adapted from [6].

The Bcl-2 family of proteins plays a major role in the regulation of apoptosis, a highly conserved programmed cell death process that eliminates unwanted cells, including damaged cells, which is essential during development [1]. Dysregulation of this process can lead to cancer or degenerative diseases [2,3] and multiple clinical trials aim to inhibit the Bcl-2 family proteins in cancer [4]. Bcl-2 is the founding member of this family that includes both apoptotic and non-apoptotic proteins. All of these actors are classified within three groups according to the presence of conserved Bcl-2 homology (BH) domains: the anti-apoptotic members that contain the four BH domains (BH1-4, including Bcl-2, Bcl-xL, and Mcl-1), the pro-apoptotic members, that lack the BH4 domain (including Bax and Bak), and the pro-apoptotic BH3-only proteins that contain the BH3 domain exclusively (including Bad, Bid, Bim) [5]. In addition, some proteins of the Bcl-2 family may contain a transmembrane domain in their C-terminus for their anchoring to intracellular membranes, including the mitochondrial outer membrane (MOM) and the endoplasmic reticulum (ER) [5]. BH1, BH2, and BH3 domains form a hydrophobic groove allowing anti-apoptotic Bcl-2 proteins to bind to the BH3 domain of apoptosis accelerators and thus inhibit cell death [6]. The interactions between all of these proteins determine the formation of “pores” in the MOM as a consequence of the homodimerization of the multi-domain apoptosis accelerators Bax and Bak, leading to the release of pro-apoptotic factors from the mitochondria, including cytochrome c (Figure 1). Cytochrome c release triggers the formation of the apoptosome, the complex responsible for the activation of caspases, the executioners of apoptosis. Caspases are cysteine proteases that under normal conditions are present in a pro-caspase inactive state and are activated by proteolytic cleavage, in turn leading to the cleavage of a large number of downstream targets, and eventually to cell death [6]. Beyond their highly documented role in apoptosis, Bcl-2 proteins and caspases have been shown to be involved in other cellular functions [5]. Notably Bcl-xL, one of the most studied anti-apoptotic Bcl-2 homolog, was reported to promote tumor stem cells properties [7], promote migration in breast cancer cells due to its effect on mitochondrial metabolism [8] and to impact intracellular calcium (Ca^2+^) trafficking by interacting with the inositol triphosphate receptor (IP3R) at the ER [9]. Bcl-xL is involved in many other non-canonical functions [5], including neurite development and growth [10]. There is evidence that impaired Bcl-x activity is correlated with the onset of major diseases such as Parkinson’s disease (PD) [11,12], amyotrophic lateral sclerosis [13] spinal cord muscular atrophy [14], as well as Friedrich ataxia [15], however the underlying molecular mechanisms remain poorly characterized. Actually, Bcl-xL seems to play critical roles in a large number of nerve cells, including astrocytes [13], neurons of the cortex [16], the hippocampus [10], the midbrain [17], the visual and the auditory systems [18,19], motoneurons (MN) [14] and sensory neurons of the dorsal root ganglion [1]. The *bclx* gene encodes 5 isoforms, including Bcl-xL, the major isoform in the CNS. The number of neurons in the CNS is determined by an equilibrium between differentiation, proliferation, and apoptosis. During the development of the CNS, axonal pruning processes take place, leading to a remodeling of neurons in order to eliminate “unnecessary” connections. These processes involve the mitochondrial apoptosis pathway, in particular the activation of caspases, leading to very localized degeneration at the subcellular level. However, during this process, the activation of caspases is independent of apoptosome formation [20]. This reflects the importance of non-apoptotic functions of the cell death machinery in the development of the CNS. This review focuses on the role of Bcl-xL and caspases downstream in the CNS with special attention to neurite outgrowth.

## 2. Mitochondria Are Crucial for Neuronal Functions and Bcl-xL Can Control Their Metabolic Activity

During the development of the CNS, mitochondria play a crucial role owing to the high levels of ATP required by nerve cells. Indeed, axon pruning, synapse formation and nerve cell plasticity are energy demanding processes. In neurons, 90% of ATP is produced by oxidative phosphorylation (OxPhos) and only 10% by glycolysis. During the development of the CNS, a metabolic switch from glycolysis to OxPhos occurs, which is critical for neuronal differentiation [21]. This switch results in a decrease in the expression of glycolytic enzymes, including lactate deshydrogenase and hexokinase [22]. The activation of ATP synthase, the enzymatic complex that produces ATP from ADP and Pi depends on the proton gradient generated by the mitochondrial electron transport chain (ETC), which takes over the electrons throughout the inner membrane of the mitochondria, resulting in H_2_O production from dioxygen (O_2_). The control of mitochondrial activity is critical in long-lived post-mitotic cells such as neurons. Indeed, neurons are polarized cells with a complex morphology, with mitochondria being transported to the exact site of ATP requirements, primarily at growth cones and at synapse formation sites. This transport is ensured by molecular motors in close interaction with microtubules and the actin microfilament network. In order to cope with these transports and related energy demands, mitochondria can undergo morphological adaptations [23]. On the one hand, mitochondrial fusion which leads to mitochondria elongation by mixing the content of damaged mitochondria, involving the mitochondrial protein Mitofusin-1 (MFN1/2), rescues defects in the mitochondrial genome via a complementation mechanism. On the other hand, mitochondrial fission, relying on the mitochondrial dynamin-related protein (DRP1), facilitates mitophagy and, therefore, the recycling of damaged mitochondria. Indeed, inhibition of mitochondrial dynamics, impairs embryonic development and is implicated in neurodegenerative diseases such as PD [24].

In neurons, mitochondrial localization of Bcl-xL seems to play a role in the mechanisms described above. First, Bcl-xL affects the synaptic localization of mitochondria by regulating mitochondrial fission through its interaction with DRP1 [25]. Interestingly, this can be compared with the remodeling of synaptic vesicles, in which the DRP1-Bcl-xL complex appears to be necessary for recapturing synaptic vesicles [26].

**Figure 2:**
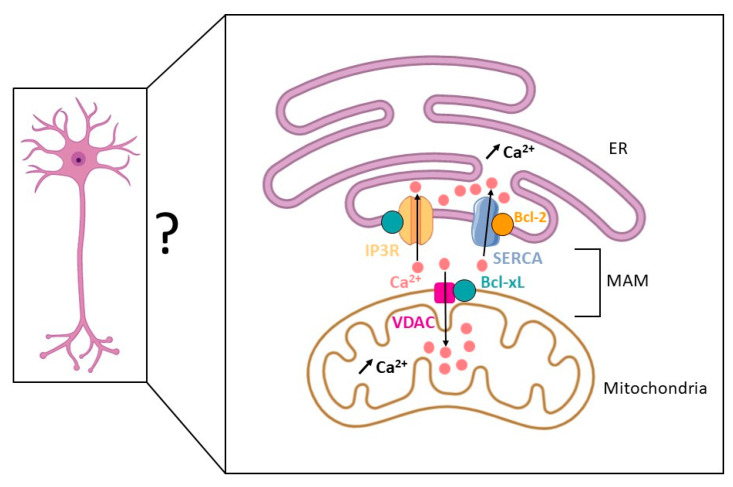
A fraction of Bcl-2 and Bcl-xL is located at the mitochondria-associated-endoplasmic reticulum membranes (MAM) to favor Ca^2+^ storage in the endoplasmic reticulum (ER) and the mitochondria. By interacting with the sarco/endoplasmic reticulum Ca^2+^ ATPase (SERCA) and inositol 1,4,5-triphosphate receptors (IP3Rs), Bcl-2 and Bcl-xL promote ER Ca^2+^ uptake. By interacting with voltage-dependent anion channel (VDAC), Bcl-xL promotes mitochondria Ca^2+^ uptake. Thus, Bcl-xL appears to play a key role in Ca^2+^ distribution at the MAM. Given the importance of Ca^2+^ dynamics in neurons, Bcl-xL contribution to Ca^2+^ homeostasis may be critical in the central nervous system (CNS).

At the synaptic level, mitochondrial Ca^2+^ uptake has an impact on the trafficking of metabolites, including ATP, between the mitochondrial matrix and the cytosol. Moreover, Bcl-xL may contribute to such trafficking, as it is able to bind to voltage-dependent anion channel (VDAC), a complex channel located at the MOM, which is critical for ADP/ATP trafficking between the mitochondria and the cytosol [27,28], see Figure 2. Indeed, VDAC permeability depends, at least in part, on interactions with Bcl-xL, which would in turn indirectly control the rate of ATP production [29]. Therefore, such interactions may contribute to the anti-apoptotic function of Bcl-xL since ATP synthesis is expected to promote neuron survival. VDAC plays a critical role regarding mitochondrial Ca^2+^ uptake. Actually a large number of mitochondrial enzymes and transporters are Ca^2+^-dependent, including enzymes of the tri-carboxylic acid (TCA) cycle (pyruvate dehydrogenase, isocitrate dehydrogenase, α-ketoglutarate dehydrogenase), ETC components, ATP synthase a well as the ADP/ATP translocator [30,31,32,33]. Thus, via its ability to bind to VDAC, which enhances mitochondrial Ca^2+^ uptake, Bcl-xL is considered as an activator of mitochondrial metabolism [34].

Furthermore, in contradiction with longstanding knowledge, Bcl-xL was recently also shown to be localized at the inner mitochondrial membrane (IMM). However, the exact fraction of Bcl-xL present at the IMM remains unknown. Nevertheless, the fact that at the IMM, Bcl-xL can interact with the ATP synthase β subunit, may allow direct control of ETC activity. Indeed, such an interaction may stabilize the mitochondrial membrane potential (∆ψ_m_), limiting ion leakage through the IMM, and increasing ATP production [35]. Moreover, in neurons this interaction may be regulated by cyclin B1 which, by phosphorylating Bcl-xL, may prevent interactions with ATP synthase. Collectively, these data provide new research avenues regarding the involvement of cyclin B1 in neurodegeneration [36].

Finally, with regard to PD, it is worth be noted that Bcl-xL was reported to interact with the PTEN-induced putative *kinase* 1(PINK-1 kinase), which may contribute to PINK-1-dependent protection against cell death [11], and to prevent the mitochondrial localization of Parkin, inhibiting in this way mitochondrial protein ubiquitination and subsequent mitophagy [12,37].

## 3. Bcl-xL and Calcium Homeostasis

Ca^2+^ is a second messenger critical for cellular physiology. Indeed, cellular Ca^2+^ dynamics contributes to major signaling pathways that controls, among other functions, cell growth and survival. Perturbations in Ca^2+^ homoeostasis can lead to variety of disorders, such as cardiovascular diseases, diabetes, tumorigenesis, and hepatic steatosis [38].

In nerve cells, Ca^2+^ plays critical roles, notably regarding synaptic transmission [39]. Given the importance of Ca^2+^-dependent events, a multiplicity of regulatory mechanisms take place to control Ca^2+^ homeostasis in neurons, including via the regulation of ER Ca^2+^ uptake [40,41].

The ER is the main Ca^2+^ storage site of the cell. SERCA plays a key role in Ca^2+^ uptake in the ER lumen by uploading free Ca^2+^ from the cytosol into the ER lumen at the expense of ATP hydrolysis [42]. Regarding Bcl-2 family proteins it has been demonstrated that Bcl-2 itself is able to bind to SERCA, resulting in SERCA inhibition and destabilization [43]. However, in another study this Bcl-2/SERCA interaction was reported to enhance SERCA activity [44]. Of note, Bcl-xL was reported to control SERCA expression, although the underlying mechanism remains unknown [45]. Despite such interactions have not been documented in nerve cells as yet, these data raise the idea that the control of SERCA activity by Bcl-2 homologs, including Bcl-xL, may be important in neurons, fostering Ca^2+^ uptake in the ER lumen and thus contributing to shape Ca^2+^ peaks during neurotransmission. In this respect, it should be noted that, in the CNS, SERCA-mediated Ca^2+^ dyshomeostasis has been associated with neurological disorders such as bipolar disorder, schizophrenia, PD, and Alzheimer’s disease (AD) [46].

Another regulator of Ca^2+^ fluxes at the ER is the IP3R family of Ca^2+^ channels (Figure 2). As mentioned above, Bcl-xL binds to IP3R Ca^2+^ channels through its BH4 domain, limiting in this way Ca^2+^ leakage from the ER to the cytosol [47].

Mitochondria is another site of Ca^2+^ storage in the cell. Indeed, at the level of the MOM, Bcl-xL was shown to bind to VDAC-1 and VDAC-3 and enhance mitochondrial Ca^2+^ uptake [34].

Furthermore, since among IP3R Ca^2+^ channels, IP3R3 appears to regulate mitochondrial Ca^2+^ signaling at the MAM [48], Bcl-xL may also contribute to Ca^2+^ homeostasis by acting on direct Ca^2+^ exchanges at the interface between the ER and the mitochondria. Indeed, a recent study by Williams and colleagues reported that the BH4 domain of Bcl-xL may act as a MAM-addressing domain through its contribution to Bcl-xL/IP3R3 interaction. Moreover, these authors showed that this interaction facilitates the redistribution of Ca^2+^ from the ER to the mitochondria, while lowering Ca^2+^ efflux to the cytosol, and promotes in this way TCA cycle activity [49]. Thus, Bcl-xL may partly reside at the MAM to play an integrative role regarding cell bioenergetics and cell survival through direct Ca^2+^ exchanges at the ER-mitochondria interface.

## 4. Bcl-xL Impacts on Neuron Development and Growth

Bcl-xL, as an anti-apoptotic factor, can prevent neuronal cell death. However, it is also involved in neuronal growth and differentiation. As mentioned above, Bcl-xL impacts synapse formation through the regulation of mitochondrial metabolism. Evidence suggests that, overall, Bcl-xL plays critical roles in sustaining neuronal differentiation, including in the context of the development of the CNS.

*Bclx* KO mice die prematurely at embryonic day E13, which is correlated with massive apoptosis in the CNS, highlighting the key role of Bcl-xL during vertebrate development [50]. Indeed, the mouse embryo, the expression of Bcl-xL begins at E11 in the spinal cord with a peak between embryonic day E13.5 and postnatal day P5 [51]. In order to better study the role of Bcl-xL during neuronal development, CNS-specific conditional *bclx* KO mice (BKO) were generated to measure the effect of *bclx* invalidation in the CNS [52]. In BKO mice, apoptosis appears at E11 on the ventrolateral side of the spinal cord, where MNs are located.

Then at E13 a peak in apoptosis occurs and spreads throughout the spinal cord. At E18, this apoptosis wave stops. Occurrence of apoptosis in BKOs is spatio-temporally correlated with neuronal differentiation, suggesting that neurons are dependent on Bcl-xL for their differentiation. More precisely, the cells that undergo apoptosis in this model are those that have exited the cell cycle and undergone differentiation. Thus, it seems that during development, Bcl-xL-dependent cells are likely post-mitotic neurons [52].

The importance of Bcl-xL in MNs was also demonstrated in a study in which MNs degeneration, resulting from a deficiency in the survival motor neuron gene (SMN) could be rescued by Bcl-xL expression [14].

In addition, another laboratory showed that, during the differentiation of neuronal progenitor cells (NPC) into immature neurons, a “dependence switch” took place, in which NPCs were dependent on Mcl-1, another Bcl-2 homolog, whereas immature neurons depended on Bcl-xL [53]. This work highlighted that although Mcl-1 and Bcl-xL have distinct BH3-only partners in this switch, both prevented Bax activation.

Furthermore, it was confirmed using conditional KO that Bcl-xL is dispensable for NPCs survival, but that specific populationof post-mitotic neurons are critically dependent on Bcl-xL for their survival. In addition, NPC-*bclx*-KO mice exhibited severe behavioral problems, such as self-mutilation and hyperactivity, suggesting that the neurons depending on Bcl-xL during development are those responsible for behavioral control [16].

The importance of Bcl-xL for neurite outgrowth could also be explained by its ability to regulate death receptor 6 (DR6) in neurons. Indeed, DR6 is known to induce axon pruning. This receptor is activated upon nerve growth factor (NGF) deprivation or under hypoxic conditions. Indeed, the NGF or oxygen deprivation leads to the cleavage of the amyloid precursor protein (APP) into N-APP, the main ligand of DR6. This subsequently induces neurite degeneration through Bax-dependent caspase-6 activation [54]. In an interesting study performed with primary neurons [10], si-RNA-mediated *bclx* silencing, as well as hypoxia-dependent caspase activation, compromised neurite outgrowth, which was associated with DR6 overexpression, thus establishing a positive feedback loop of DR6 activation following its own expression. Moreover, depletion of DR6 was sufficient to suppress this effect, confirming that DR6 is under the control of Bcl-xL. According to this model, during hypoxia, Bcl-xL may protect neurons from the effect of DR6 activation, presumably by preventing Bax oligomerization.

## 5. Caspases Regulate Neuronal Plasticity

Though its ability to prevent pores formation and cytochrome c release, Bcl-xL regulates caspases activation. During neurodevelopment, caspases participate in programmed cell death processes to eliminate excess neurons, which is critical for full maturation of the CNS. There is evidence that caspase-3 regulates such developmental neural cell death, allowing to stabilize proper synaptic contacts [55]. However, caspases have also reported in the modulation of neuronal plasticity, independently of their apoptotic role. Indeed, during neuronal development, dendrite outgrowth and axon pruning require cytoskeletal remodeling to allow morphological changes. The reorganization of the cytoskeleton depends on the balance between polymerization and depolymerization, which notably relies on proteolytic activities. Indeed, it was demonstrated that caspases, in particular the effector caspases-3 and -6, can cleave microtubule and microfilament components in the context of axonal degeneration, including axon pruning [56]. Moreover, in a study investigating the role of the ubiquitin E3 ligase E6AP in autism, it was shown that E6AP expression activates caspase-3 through the inhibition of the X-linked inhibitor of apoptosis (XIAP) responsible for caspase-3 ubiquitination, leading to microtubule cleavage and disorganization of axonal arborization [57]. These observations highlight the fact that caspase activation must be finely regulated not to be detrimental.

Besides cytoskeleton components, additional substrates of caspases have been identified in neurons. Among them, microtubule-associated proteins (MAP) such as MAP-2, dyneins, and dynamins family proteins have been identified as caspase-3 substrates by proteomic studies [58]. Caspase-3 has also been reported to cleave growth associated protein 43 (GAP43). Indeed, cleaved GAP43 allows endocytosis of the α-amino-3-hydroxy-5-methyl-4-isoxazolepropionic acid (AMPA) receptor in the context of long-term depression (LTD) [59]. N-ethylmaleimide sensitive fusion protein (NSF) involved in synaptic vesicle fusion with the cell membrane and synaptin involved in neurotransmitter release are other targets of caspase-3 in neurons [58]. Collectively, these observations support the idea that caspase-3 play major roles in neurons, notably at the synapse level.

The precise effect of caspases on the neuronal network is not yet fully elucidated, but it seems that caspase activation may favor the “pathfinding mode”, which results in retraction of terminal axons and sprouting of new branches, over the “targeting mode”, which results in elongation of terminal axons. Indeed, a dominant negative caspase-3 mutant was shown to increase the length of the terminal branch and decrease the number of branch points in the ciliary ganglia of chicken embryos [60].

Therefore, during development, caspases appear to trigger axon degeneration either in the context of axon pruning or during apoptosis. However, despite this overlap, the regulation of both processes seems to be independent. Indeed, in a study using microfluidic chambers to induce local NGF or global NGF deprivation, to, respectively, decipher local degeneration and apoptosis, depletion of caspase-6, which has been previously described as being involved in axonal degeneration [61], was able to block axonal degradation when local NGF deprivation but unable to do so when global deprivation [62]. Together, these results suggest that neurons have caspase 6-dependent and caspase 6-independent pathways to trigger axonal degeneration and that their activation depends on the location of trophic factors withdrawal.

An additional study conducted with mouse olfactory neurons, highlighted the non-apoptotic role of caspase-9. This caspase is capable of cleaving semaphorin 7A, a guidance molecule required for dendrite projections. Indeed, the authors observed maturation problems of olfactory neurons, misrouted axons and defects in synapse formation in caspase9 KO mice. Therefore, caspase-9 seems to participate in the building of the olfactory neuronal network [63]. This result also raises the notion that caspase activity fosters pathfinding during axon pruning so that each neuron can find its target and form the appropriate synapse. To avoid excessive caspase activation and subsequent apoptosis, it is assumed that this activation is localized and transient. It has been proposed that the local and transient activation of caspases in dendrites is controlled by proteasome activity, on the one hand, and by the ubiquitin ligase activity of the inhibitors of apoptosis proteins (IAP), on the other hand [64].

The importance of caspases in the neuronal system has not only been described during development but also during synaptic modification processes in adults. LTD is an essential process for synaptic plasticity that involves the activation of N-méthyl-D-aspartate (NMDA) receptors and the internalization of AMPA receptors contributing to synaptic transmission. A study showed that caspase-3 inhibition significantly decreased LTD [65]. According to this model, in hippocampal neurons, NMDA receptor activation triggered transient caspase-3 activity, and promoted LTD without cell death. It should be noted that another study in PD supports this model, showing a decrease in LTD associated with decreased caspase-3 activity [66].

## 6. Bcl-x Caspase Crosstalk

The present review highlights the role of both Bcl-x and caspases on the maturation of the nervous system. Actually, numerous studies have reported that Bcl-xL acts on caspase status and vice versa. Indeed, such crosstalks must be considered in a complete description of thecell growth and differentiation mechanisms depending both on Bcl-2 proteins and the caspase family of proteases.

Bcl-xL has long been known to prevent mitochondria-dependent caspase activation, whereas the Bcl-xS isoform has the opposite effect [67]. Thus, the balance between Bcl-x isoforms through alternative splicing might be taken into account regarding CNS maturation. In addition, it should be recalled that Bcl-xL can be cleaved by caspase-3 and converted to an apoptosis accelerator [68]. The contribution of such an event to neurite outgrowth has not been fully evaluated.

Overall, Bcl-x caspase intertalk may be instrumental in the tuning of caspase activity such that maturation processes can be achieved without apoptosis induction, which might be deleterious [69].

The respective distribution of above actors is another interesting issue. Indeed, it may be critical to prevent mitochondria-dependent apoptosis, and thus caspase activation, in the soma, whereas caspases are activated at the same time in axons via extrinsic pathways to ensure elongation and branching [54]. In this respect there is evidence that Bcl-xL participates in the remote control of axon degeneration by the soma [70].

## 7. Concluding Remarks

The findings presented in this review may seem unexpected, since major differentiation events in nerve cells (well-known for their extremely long lifespan) including axonal growth, depend, at least in part, on the apoptosis machinery. This is a perfect illustration of Nature’s tendency to divert a number of proteins from their original role to ensure “moonlighting” functions.

**Figure 3:**
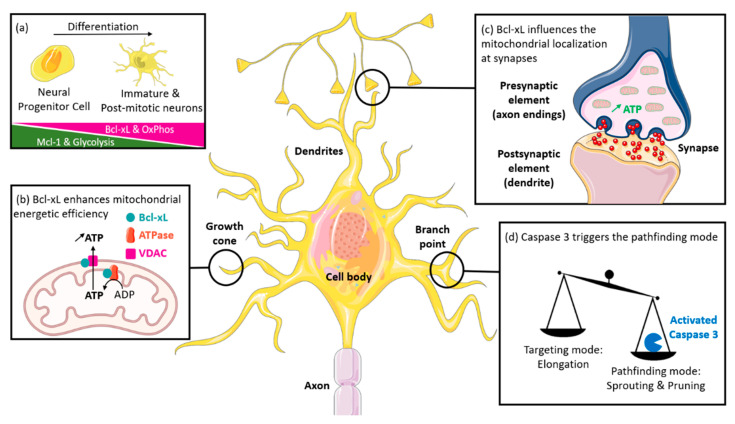
Main roles of Bcl-xL and caspase-3 in the nervous system. (**a**) During the development of the nervous system, neuronal progenitor cells (NPC) differentiate into immature neurons that may complete their differentiation and become post-mitotic and fully mature. This differentiation is associated with a metabolic switch from glycolysis to oxydative phosphorylation (OxPhos) and seems to be dependent on the expression of Bcl-xL. In differentiated neurons (**b**) mitochondria-addressed Bcl-xL, boosts the bioenergetics of the cell, by interacting with ATP synthase, which is critical for neurite outgrowth, and (**c**) allows the recruitment of mitochondria at the synaptic level via an unknown mechanism, which has a positive effect on synapse formation and function, including synaptic vesicle formation. (**d**) Caspase-3, a major apoptosis executioner, is required for the maturation of the nervous system. Indeed, caspase-3 promotes axonal branching so that neurites find their path to the proper target.

We herein presented the key involvement of the Bcl-xL anti-apoptotic protein in neuronal growth and differentiation through mechanisms related or not to its canonical anti-apoptotic role. Differentiated neurons become progressively dependent on Bcl-xL (Figure 3a) as mitochondria-addressed Bcl-xL is required for dendrite growth (Figure 3b) and synapse formation (Figure 3c).

However, it would also be relevant to investigate the role of ER-addressed Bcl-xL, given its ability to regulate Ca^2+^ fluxes by regulating the permeability of ER-anchored Ca^2+^ channels. Indeed, since neurotransmission involves Ca^2+^ fluxes that need to be highly regulated to avoid damages, ER-localized Bcl-xL presumably plays a protective role against Ca^2+^ toxicity in firing neurons. Thus, in nerve cells, depending on its subcellular localization, Bcl-xL presumably ensures distinct though complementary functions.

We also reviewed the role of caspases, notably caspase-3, the major apoptosis effector in the development of the CNS. Caspase-3 was reported to be involved in neuron plasticity, presumably through its ability to contribute to cytoskeletal remodeling.

Indeed, caspase-3 promotes sprouting and pruning of dendrites (Figure 3d). To achieve this function, its activation is transient and takes place exclusively in dendrites. As a result, caspase-3 can modulate cell plasticity without causing cell death. It would be relevant to investigate the role of Bcl-xL in this spatio-temporal regulation. Of note, caspase-3 activation was described in AD at the post-synaptic level and co-localized with Aβ senile plaques. However, the significance of these observations remains unclear [71]. Therefore, a better understanding of the roles of caspase-3 and Bcl-xL in neurons might have future clinical consequences, including in the field of neurodegenerative diseases.
